# Antioxidant and ACE Inhibitory Bioactive Peptides Purified from Egg Yolk Proteins

**DOI:** 10.3390/ijms161226155

**Published:** 2015-12-07

**Authors:** Marwa Yousr, Nazlin Howell

**Affiliations:** Faculty of Health and Medical Sciences, the University of Surrey, Guildford, Surrey GU2 7XH, UK; marwa_yousr@yahoo.co.uk

**Keywords:** egg yolk peptides, antioxidant activity, DPPH (1,1-Diphenyl-2-Picrylhydrazyl), hydroxyl radical, chelating activity, malondialdehyde, ACE inhibition

## Abstract

Protein by-products from the extraction of lecithin from egg yolk can be converted into value-added products, such as bioactive hydrolysates and peptides that have potential health enhancing antioxidant, and antihypertensive properties. In this study, the antioxidant and angiotensin converting enzyme (ACE) inhibitory activities of peptides isolated and purified from egg yolk protein were investigated. Defatted egg yolk was hydrolyzed using pepsin and pancreatin and sequentially fractionated by ultrafiltration, followed by gel filtration to produce egg yolk gel filtration fractions (EYGF). Of these, two fractions, EYGF-23 and EYGF-33, effectively inhibited the peroxides and thiobarbituric acid reactive substance (TBARS) in an oxidizing linoleic acid model system. The antioxidant mechanism involved superoxide anion and hydroxyl radicals scavenging and ferrous chelation. The presence of hydrophobic amino acids such as tyrosine (Y) and tryptophan (W), in sequences identified by LC-MS as WYGPD (EYGF-23) and KLSDW (EYGF-33), contributed to the antioxidant activity and were not significantly different from the synthetic BHA antioxidant. A third fraction (EYGF-56) was also purified from egg yolk protein by gel filtration and exhibited high ACE inhibitory activity (69%) and IC_50_ value (3.35 mg/mL). The SDNRNQGY peptide (10 mg/mL) had ACE inhibitory activity, which was not significantly different from that of the positive control captopril (0.5 mg/mL). In addition, YPSPV in (EYGF-33) (10 mg/mL) had higher ACE inhibitory activity compared with captopril. These findings indicated a substantial potential for producing valuable peptides with antioxidant and ACE inhibitory activity from egg yolk.

## 1. Introduction

Scientific research has focused heavily on evaluating the nutritional value of the hen’s eggs. In addition to the high nutritional value, egg yolk is an important source of lecithin, which is widely used as a surfactant, a lubricant and as an emulsifying agent in pharmaceutical and industrial applications [1]. The waste products that result from the extraction of lecithin from egg yolk are potentially valuable by-products as they contain a substantial amount of protein, which represents approximately 30% of dried egg yolk. This protein could be converted into value-added bioactive peptides by enzymatic hydrolysis; however, there are very few reported studies on the utilization of the egg yolk protein by-product. Bioactive peptides usually contain 2–20 amino acid residues per molecule, and are released upon enzymatic hydrolysis, during food processing or during gastrointestinal digestion [2]. These peptides may exert diverse physiological effects, such as antihypertensive, antimicrobial, antithrombotic, hypocholesterolaemic, and antioxidant actions [2,3,4,5] due to the amino acid composition, sequence, and molecular weight [6]. In this paper, two aspects of the diverse function of bioactive peptides were studied to enhance nutritional properties of food, *i.e.*, by improving the preservation and nutritional value by reducing lipid oxidation and also by enhancing health benefits by acting as a potential angiotensin converting enzyme (ACE) inhibitor.

In food systems, lipid oxidation via free radicals can alter organoleptic properties, giving rise to unacceptable taste, flavor, and rancidity, as well as toxic compounds that reduce the quality and the shelf life of products [7]. Therefore, it is important to inhibit or minimize lipid oxidation in food processing by using antioxidants [8]. Although synthetic antioxidants show strong activity in inhibiting the deterioration of lipids during storage, there is a concern about their toxicity and carcinogenicity at high levels of intake [9,10]; therefore, natural antioxidants have recently been a focus of research [11]. In this context, protein hydrolysates and peptides prepared from food proteins have emerged as a new source of natural antioxidants. For example, sunflower protein [12], casein [13], soybean protein [14], egg-white albumen [15], and Pacific hake protein [16] have all been identified as potential sources of antioxidant peptides, demonstrating strong antioxidant activity through different pathways, including radical scavenging and metal ion chelating activity. Several studies have reported antioxidant peptides from egg, including egg albumen and egg yolk proteins [17,18,19]. In terms of egg yolk proteins, which the present study is related to, isolation of antioxidant peptides from lecithin-free egg yolk using industrial proteases, or a food-grade proteinase from *Bacillus* sp. [20] showed good free radical scavenging properties. Sakanaka *et al.* [21,22] hydrolyzed fat free egg yolk proteins using orintase and protease, and only the antioxidant activity of the hydrolysate was tested, not the purified fractions. Enzyme specificity and degree of hydrolysis are some of the important factors that affect bioactivity when peptides are prepared *in vitro*. While most studies related to purifying bioactive peptides have employed microbial proteases, such as those involved in milk processing, only one study on phosvitin has been reported [23] to use enzymes involved in human physiological digestion; therefore, this is a novel study using digestive enzymes pepsin and pancreatin to hydrolyze whole egg yolk protein.

Bioactive peptides from food proteins have also exhibited ACE inhibitory activity. High blood pressure is a chronic medical symptom leading to worldwide health problems because of its ability to trigger cardiovascular complications including peripheral vascular disease and renal dysfunction. About one billion people suffer from hypertension, resulting in over 7.1 million deaths per year globally [24]. In the UK, the number of people diagnosed with hypertension rose by 2.7% from 2004 to 2008 [24].

Regulation of arterial blood pressure in the human body is mainly achieved through diverse physiological systems including the kinin-nitric oxide system (KNOS), the neutral endopeptidase system (NEPS), the renin-chymase system (RCS), and the renin-angiotensin system (RAS). One of the key elements constituting the renin-angiotensin system is the angiotensin converting enzyme (ACE; EC3.4.15.1, dicarboxy peptidase). ACE hydrolyzes inactive decapeptide (angiotensin-I), by the removal of the dipeptide His-Leu from the C-terminus, to produce the potent vasoconstrictor octapeptide (Angiotensin-II) [25]. Angiotensin-I results from the cleavage of angiotensinogen (Asp-Arg-Val-Tyr-Ile-His-Pro-Phe-His-Leu-Val-Ile-His-Glu-Ser) from the liver by the action of renal renin. ACE enhances the reabsorption of renal tubular sodium by increasing the release of adrenal aldosterone. ACE also hydrolyses vasodilatory bradykinin to inactive metabolites in the depressor hormonal (kinin-kallikrein) system [26]. Therefore, ACE inhibitors block the generation of vasoconstrictor angiotensin II and potentiate the action of the vasodilator bradykinin.

Although potent synthetic ACE inhibitors namely captopril, lisinopril, fosinopril, and enalapril are used extensively in the clinical treatment of hypertension, they have significant adverse effects on health such as a dry cough, skin rashes, and headache [27]. Recent studies on diverse peptides from whey [28], egg [29,30] and soy proteins [31] have shown ACE inhibition. Studies on food bioactive peptides do not indicate side effects in humans [32]. Accordingly, more studies are now directed towards understanding how to produce food-derived peptides to be used as nutraceuticals for lowering hypertension.

The objectives of the present study were: (1) to produce efficient bioactive peptide fractions from egg yolk protein (EYP) by digestion with physiological proteases followed by purification using a range of ultrafiltration and chromatographic separations; (2) to investigate antioxidant activities and mechanisms in different model systems; (3) to assess the ACE inhibitory activity; and (4) to characterize the fractions by amino acid analysis and identify peptide sequences.

## 2. Results and Discussion

### 2.1. Protein Extraction and Quantification

Defatting of egg yolk with hexane and ethanol resulted in a white powder. The lipid was removed successfully resulting in a high percentage of protein, as determined by the Kjeldahl method. Protein content of the defatted egg yolk sample was 91.0% ± 0.2% compared with native egg yolk 30.2% ± 0.2.%.

### 2.2. Egg Yolk Protein Hydrolysis and Ultrafiltration

A combination of two enzymes pepsin and pancreatin not only mimicked the digestive processes in the human body, but also achieved a higher degree of hydrolysis than that produced when using either pepsin or pancreatin alone. Chen *et al.* [33] reported that the antioxidant potential of protein hydrolysates can be enhanced with specific enzymes and optimal hydrolysis conditions. Accordingly, many studies have been conducted using enzymatic hydrolysis to improve the functional properties and activity of isolated proteins [33,34].

Antioxidative peptides from egg yolk protein hydrolysates (EYPH), were fractionated by passing sequentially through three ultrafiltration (UF) membranes with molecular weight cut-offs (MWCOs) of 10, 5, and 2 kDa (that allowed molecules below the selected molecular size to pass through) and resulted in ultrafiltered hydrolysate fractions called EYUF-10 (≤10 kDa), EYUF-5 (≤5 kDa) and EYUF-2 (≤2 kDa). EYUF-2 represented the highest yield recovery 70% ± 2.1% followed by 12% ± 1.2% for EYUF-5 and 10% ± 1.1% for EYUF-10 peptides. The high yield of the smaller molecular weight fraction, obtained from ultrafiltration process (EYUF-2), indicated that egg yolk protein (EYP) was hydrolysed extensively. The protein content of the purified ultrafiltered fractions was 96% ± 0.2% which was used for further experiments.

### 2.3. Purification of Antioxidative Peptides

#### 2.3.1. Measurement of Lipid Oxidation Inhibition Activity of Ultrafiltration Fractions

**Figure 1:**
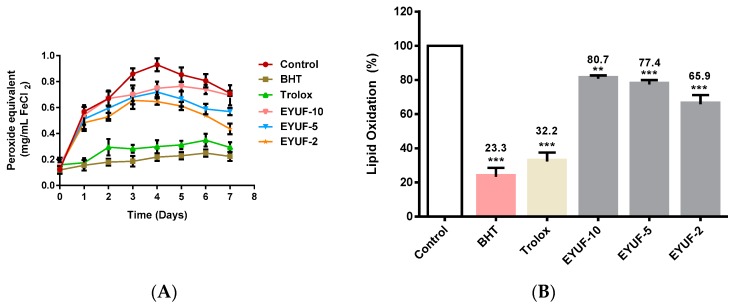
Effect of fractionated egg yolk protein hydrolysates on lipid oxidation by ferric thiocyanate (FTC) method. Lipid oxidation was measured in a linoleic acid model system. (**A**) Peroxide concentration was monitored every 24 h for 7 days at 500 nm; (**B**) The percentage of lipid oxidation, indicated at the top of the bar, was measured after four days incubation at 40 °C. BHT and trolox (0.2 mg/mL of each), shown in colour, were used as positive controls, while milli-Q water was used in the control instead of sample. EYUF-10, EYUF-5 and EYUF-2 fractions were collected after passing through 10, 5, and 2 kDa ultrafiltration membranes (50 mg/mL of each fraction). Data correspond to the means ± SD of three independent experiments. ANOVA was performed in Graphpad Prism version 6.0, followed by Dunnett’s multiple comparisons test. The result was considered statistically significant *versus* the water control (** = *p* < 0.01, *** = *p* < 0.001). BHT: butylated hydroxyltoluene; EYUF: Egg yolk ultrafiltered fraction.

The linoleic acid oxidizing model system verified the oxidation inhibition activity of fractionated egg yolk protein hydrolysate (EYPH), using the ferric thiocyanate (FTC) and thiobarbituric acid reactive species (TBARS) methods and was used to select the most effective peptide fractions for further purification. In this study, the antioxidant effect of three fractions from EYPH was investigated and compared with trolox and butylated hydroxyltoluene (BHT) as a synthetic antioxidant. With the FTC method, samples incubated with linoleic acid in the model system at 40 °C produced peroxides that were monitored for 7 days as shown in Figure 1A. Peak peroxide concentrations were detected on day 4 in all samples, therefore, the percentage of lipid oxidation was estimated at this time point (Figure 1B). All fractions significantly decreased the percentage of lipid oxidation when compared to the negative control (all at least *p* < 0.01). Lipid oxidation (%) in the presence of EYUF-2, EYUF-5 and EYUF-10 was 65.9%, 77.4% and 80.7% respectively. BHT and trolox also significantly decreased the percentage of lipid oxidation to 23.3% and 32.2%, respectively, when compared to the negative control (*p* < 0.001).

**Figure 2:**
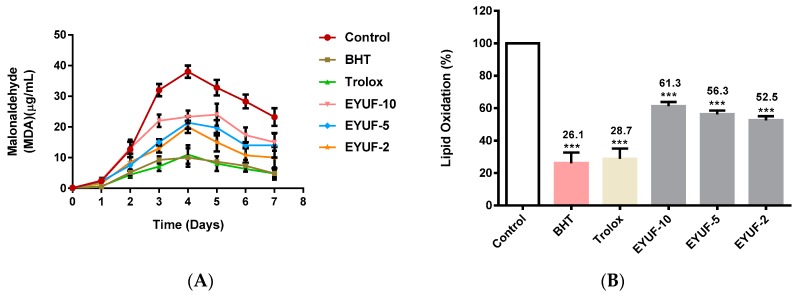
Effect of fractionated egg yolk protein hydrolysates on lipid oxidation by thiobarbituric reactive substance (TBARS) method. (**A**) Malondialdehyde concentration was monitored every 24 h for 7 days at 532 nm; (**B**) Percentage of lipid oxidation, indicated at the top of the bar, was measured after four days incubation at 40 °C. BHT and trolox (0.2 mg/mL of each), shown in colour, were used as positive controls, while milli-Q water was used in the negative control instead of sample. EYUF-10, EYUF-5 and EYUF-2 are fractions collected after passing through 10, 5, and 2 kDa ultrafiltration membranes (50 mg/mL of each fraction). Data correspond to the means ± SD of three independent experiments. ANOVA was performed in Graphpad Prism version 6.0, followed by Dunnett’s multiple comparisons test. The result was considered statistically significant *versus* the water control (*** = *p* < 0.001).

In the TBARS assay, samples were incubated with linoleic acid in the model system at 40 °C and formation of malonaldehyde (MDA) was monitored for 7 days as shown (Figure 2A). Maximal concentrations of MDA were measured on day 4, which formed the basis of this time-point selection for investigation of percent lipid oxidation (Figure 2B). The percentage of lipid oxidation was significantly decreased in the presence of EYUF-2 followed by EYUF-5 and EYUF-10 when compared to the negative control (all *p* < 0.001; Figure 2B). Among the three fractions, the lowest lipid oxidation was observed for the 2 kDa fraction (52.5%) followed by 5 kDa (56.3%) then 10 kDa (61.3%). The presence of BHT and trolox in a linoleic acid model system significantly inhibited lipid oxidation to 26.1% and 28.7%, respectively, when compared to the negative control (*p* < 0.001 for both; Figure 2B).

The highest activity in both methods FTC and TBARS was highlighted by the smallest molecular weight fraction EYUF-2. This finding may relate to the small molecular weight of the peptides in this fraction, as many studies have shown that peptides of smaller molecular weight exhibit greater antioxidative activity [28,29,30,31,32,33,34]. Therefore, only the EYUF-2 fraction with the highest antioxidative activity was chosen for further fractionation.

#### 2.3.2. Measurement of Lipid Oxidation Inhibition Activity of Purified Peptide after Gel Filtration

**Figure 3:**
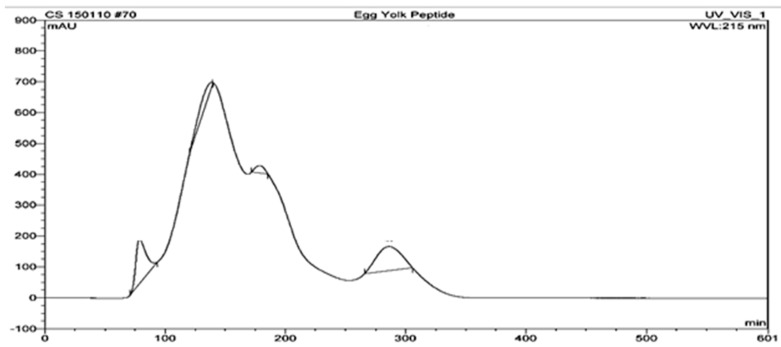
Elution profile of EYUF-2 separated by gel permeation chromatography (GPC) on a Sephadex G-25 column. Separation of peptides was detected at 215 nm with flow rate of 1 mL/min.

The EYUF-2 fraction with the highest antioxidant activity, collected from the ultrafiltration process and separated by gel filtration chromatography using a Sephadex G-25 column, resulted in more purified peptides. The elution profile generated with a flow rate of 1 mL/min is shown in Figure 3.

**Figure 4:**
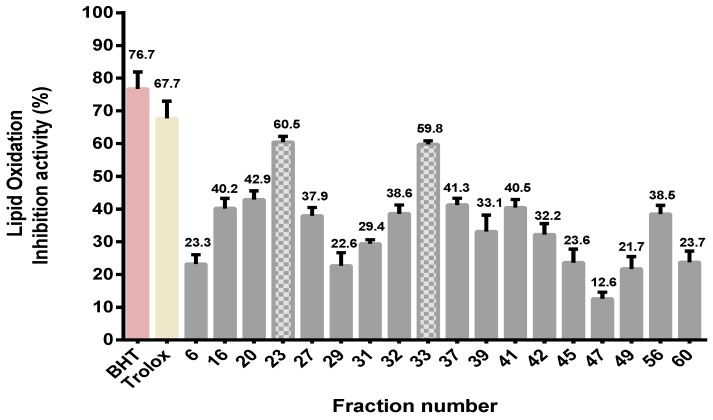
Lipid oxidation inhibition activity (%) is shown at the top of the bars for gel filtration fractions using the ferric thiocyanate (FTC) method. The activity was analyzed for 50 mg/mL of all fractions separated on a Sephadex G-25 column. The absorbance was measured after 4 days incubation at a wavelength of 500 nm. Data correspond to the means ± SD of three independent experiments. The results were compared with positive controls (0.2 mg/mL) trolox and (0.2 mg/mL) BHT, shown in colour. The two shaded bars (fractions 23 and 33) showed the highest activities and were selected for further study.

Fractions obtained from gel permeation chromatography (GPC) were pooled, then lyophilized and their oxidation inhibition activity was assayed using the FTC method. The method was conducted as a primary overview of all fractions to test their ability to inhibit lipid oxidation when used at a concentration of 50 mg/mL. Figure 4 shows that lipid oxidation inhibition activity was widely observed for all gel filtration fractions (EYGF), but the most effective fractions depicted by shaded bars were EYGF-23 (60.5% inhibition) followed by EYGF-33 (59.8% inhibition). These two fractions were, therefore, collected for further study.

### 2.4. Antioxidant Activities of Egg Yolk Peptides

In order to understand the mechanism of antioxidant action of the peptide fractions, many assays were used including DPPH (1,1-Diphenyl-2-Picrylhydrazyl) radical scavenging, hydroxyl radical scavenging, superoxide anion scavenging and iron chelating activities.

DPPH radical scavenging activity of EYGF-23 and EYGF-33 increased with concentration in a dose-dependent manner. When comparing the scavenging activity of EYGF-23 and EYGF-33 with trolox, all the concentrations used from both fractions were significantly lower than trolox (38.5%, *p* < 0.05). In contrast, only the highest concentration of both fractions (20 mg/mL) exhibited significantly higher scavenging activity when compared with BHT (23.6%, *p* < 0.05). The hydroxyl radical scavenging activity (HRSA) of EYGF-23 and EYGF-33 as well as superoxide anion scavenging activity increased with the concentration in a dose-dependent manner (Table 1A). Radical quenching activity by antioxidant peptides is considered to be one of the most effective pathways in the prevention of lipid peroxidation propagation reactions. This activity is, therefore, very important as a means of protecting biological systems against various diseases and to protect food systems from deterioration. There is a structure–function relationship between the amino acid composition and the scavenging activity of the isolated peptide. The low molecular weight of the isolated peptide, the presence of specific amino acid residues such as tryptophan and tyrosine, and their sequence within the peptide are noticeably correlated with radical scavenging activity of the peptides [35,36].

In the current study, EYGF-23 and EYGF-33 fractions both revealed the ability to act as ferrous chelating agents although the activity was significantly lower when compared with EDTA at the concentrations used (Table 1B). Ferrous ions can act as catalysts, which enhance the generation of free radicals and subsequently initiate the oxidative chain reactions. Therefore, chelating agents may result in reduced availability of these ions and inhibit the oxidative chain reactions. Chen *et al.* [37] demonstrated that the sequence of amino acids also plays an important role in the metal chelating activity of isolated peptides from soybean. The degree of peptide hydrolysis, the type of amino acid and their sequence in the isolated peptides have all been shown to alter chelation activity [35,36].

### 2.5. Purification of Angiotensin Converting Enzyme (ACE) Inhibitory Peptide

**Figure 5:**
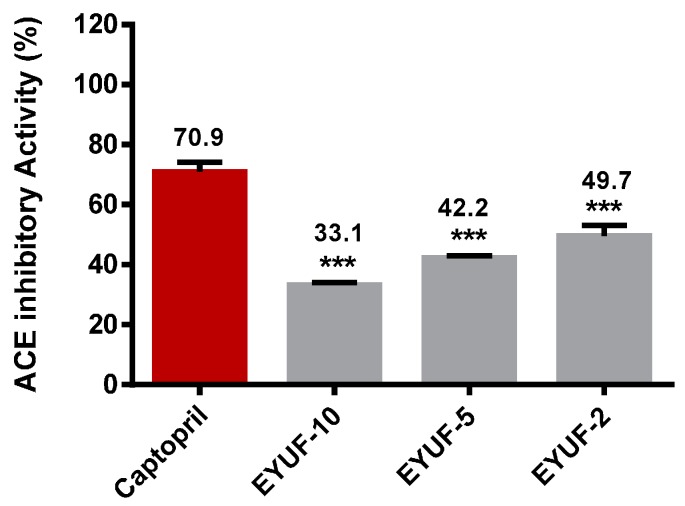
ACE inhibitory activity (%) of hydrolyzed and fractionated egg yolk protein. The activity (%) of 10 mg/mL EYUF-10, EYUF-5 and EYUF-2 to inhibit ACE was measured by HPLC and is shown at the top of the bars. Captopril (0.5 mg/mL), shown in red, was used as a positive control. Data correspond to the means ± SD of three independent experiments. ANOVA was performed in Graphpad Prism version 6.0, followed by Dunnett’s multiple comparisons test. The result was considered statistically significant *versus* captopril (*** = *p* < 0.001).

ACE inhibition was measured by quantification of hippuric acid (HA), which appears at 228 nm as a reaction product of the ACE enzyme on the substrate hippuryl histydyl leucine (HHL). The ACE inhibitory activity exhibited by 10 mg/mL of ultrafiltration fractions obtained after hydrolysis (EYUF-10, EYUF-5 and EYUF-2) was compared with that of 0.5 mg/mL captopril (Figure 5). Among the fractions, the highest inhibitory activity was observed in the EYUF-2 sample, followed by EYUF-5, then EYUF-10 (49.7%, 42.2%, and 33.1% inhibition, respectively). Inhibitory activity increased with decreasing fraction molecular weight, although none was as potent as captopril (70.9%; *p* < 0.001). The observation that ACE inhibitory activity increases with lower molecular weight concurs with many studies in this field [36,38].

**Figure 6:**
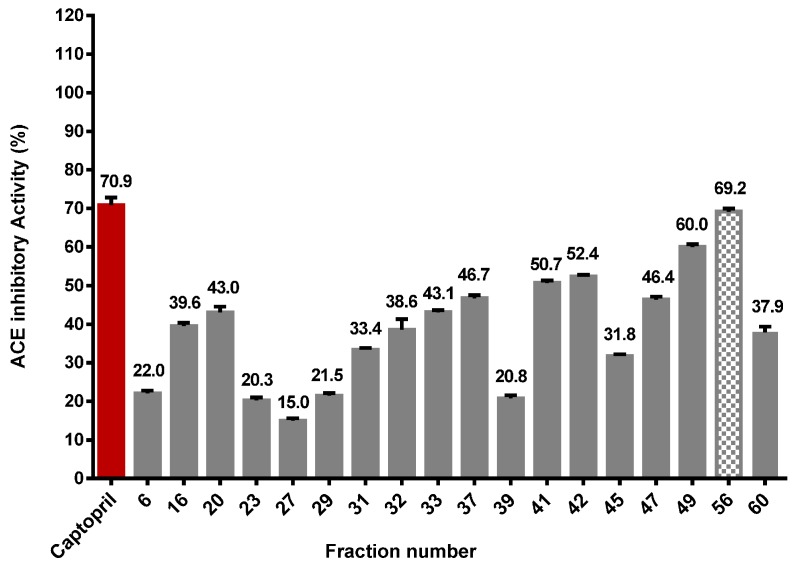
ACE inhibitory activity (%) of egg yolk protein fractions separated on a Sephadex G-25 column. The activity (%) of 10 mg/mL for each gel filtration fraction to inhibit ACE was measured by HPLC and is shown at the top of the bars. Captopril (0.5 mg/mL), shown in red, was used as a positive control. The shaded bar denotes fraction 56 with the highest ACE inhibitory activity. Data correspond to the means ± SD of three independent experiments. ANOVA was performed in Graphpad Prism version 6.0. followed by Dunnett’s multiple comparisons test.

The EYUF-2 fraction with the highest ACE inhibitory activity was collected from the ultrafiltration process and separated by gel filtration chromatography. Fractions obtained from different runs of gel filtration chromatography were pooled and lyophilized before their ACE inhibition activity was assayed. Figure 6 shows that ACE inhibition activity was widely observed for all fractions, but the most effective fraction (depicted by a shaded bar) was EYGF-56 (69.2% inhibition) compared with 0.5 mg/mL captopril.

**Table 1:** Antioxidant activities of EYGF-23 and EYGF-33. The activity was measured over a concentration range of 0.5 to 20 mg/mL for each isolated fraction (**A**) represents the activities measured using DPPH radical scavenging, HRSA and superoxide anion scavenging activity assays. The activity of both fractions was compared to trolox as a natural antioxidant standard and BHT as a synthetic antioxidant standard (both 0.2 mg/mL); (**B**) represents the chelation activity measured using the Fe^2+^ chelating activity assay, the activity was compared to EDTA (0.2 mg/mL). Data correspond to the mean ± SD of three independent experiments. ANOVA was performed in Graphpad Prism version 6.0, followed by Dunnett’s multiple comparisons test. Values with subscript a indicate significant differences with trolox, and subscript b indicate significant differences with BHT *p* < 0.05.

**(A)**		**Sample Concentration (mg/mL)**					**Trolox ^a^**	**BHT ^b^**
		**0.5**	**1**	**5**	**10**	**20**	**0.2 mg/mL**	**0.2 mg/mL**
DPPH Scavenging activity	EYGF-23	8.10% ± 1.43% ^a,b^	11.32% ± 1.05% ^a,b^	15.61% ± 2.30% ^a,b^	19.40% ± 1.10% ^a^	28.46% ± 2.13% ^a,b^	38.46% ± 1.43%	23.60% ± 1.66%
	EYGF-33	6.15% ± 1.15% ^a,b^	10.99% ± 1.90% ^a,b^	13.58% ± 1.16% ^a,b^	16.47% ± 1.49% ^a,b^	30.65% ± 2.86% ^a,b^		
Hydroxyl radical scavenging activity	EYGF-23	5.62% ± 2.33% ^a,b^	10.575% ± 1.36% ^a,b^	26.05% ± 2.86% ^a,b^	37.15% ± 2.15% ^a,b^	62.37% ± 2.21% ^a^	70.03% ± 3.48%	58.97% ± 1.02%
	EYGF-33	5.08% ± 1.44% ^a,b^	12.66% ± 1.74% ^a,b^	26.78% ± 1.57% ^a,b^	42.50% ± 2.45% ^a,b^	69.28% ± 3.62% ^b^		
Superoxide anion scavenging activity	EYGF-23	11.63% ± 2.32% ^a,b^	18.12% ± 0.51% ^a,b^	30.62% ± 1.86% ^a,b^	41.01% ± 1.70% ^a,b^	67.66% ± 2.14%	63.48% ± 1.63%	67.45% ± 1.67%
	EYGF-33	11.59% ± 1.70% ^a,b^	28.13% ± 2.77% ^a,b^	35.43% ± 3.90% ^a,b^	48.85% ± 3.14% ^a,b^	82.88% ± 2.49% ^a,b^		
(**B**)		**Sample Concentration (mg/mL)**					**EDTA ^a^**	
		**0.5**	**1**	**5**	**10**	**20**	**(0.2 mg/mL)**	
EYGF-23		12.69% ± 1.15% ^a^	20.32% ± 1.79% ^a^	32.45% ± 1.84% ^a^	64.05% ± 1.49% ^a^	94.01% ± 1.40% ^a^	97.54% ± 1.79%	
EYGF-33		10.24% ± 0.99% ^a^	24.39% ± 0.94% ^a^	41.37% ± 0.98% ^a^	61.88% ± 1.41% ^a^	92.22% ± 1.66% ^a^		

EYGF: egg yolk gel filtration fractions; HRSA: hydroxyl radical scavenging activity; BHT: butylated hydroxyltoluene; DPPH: 1,1-Diphenyl-2-Picrylhydrazyl.

The 50% enzyme inhibitory concentration (IC_50_) of the fractions with the highest ACE inhibition activity obtained from each purification step was calculated. ACE inhibition activity was enhanced with further purification; EYUF-2 exhibited the lower activity (49.7%) with an IC_50_ of 5.44 mg/mL compared with EYGF-56 which exhibited 69.2% activity and IC_50_ of 3.35 mg/mL. This observation is in agreement with previous studies reporting that purification decreased IC_50_ values [39,40].

### 2.6. Amino Acid Analysis and Sequencing

**Figure 7:**
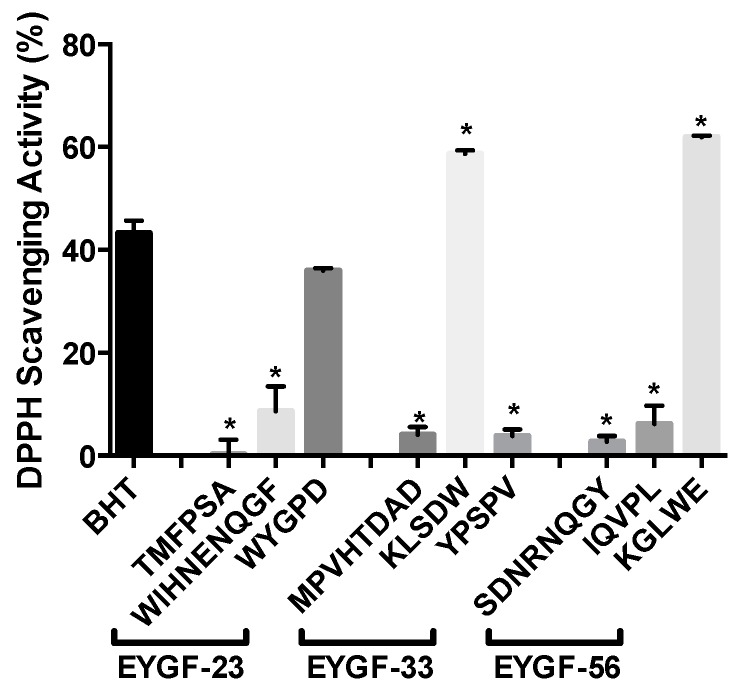
DPPH scavenging activity (%) of selected peptides. The activity was analyzed for 10 mg/mL of all isolated peptides. Data correspond to the means ± SD of three independent experiments. The results were compared with (0.2 mg/mL) butylated hydroxytoluene (BHT). The result was considered statistically significant *versus* BHT (* = *p* < 0.05).

Due to the substantial inhibitory effects of EYGF-23 and EYGF-33 on peroxide and MDA formation and the inhibitory effect of EYGF-56 on ACE activity, these fractions were subjected to amino acid analysis and LC-MS in order to elucidate the possible effect of amino acid composition and sequences on the activity shown. According to the amino acid profile (Table 2), EYGF-23 fraction contained a large proportion of proline (8.0%), positively charged lysine (11.0%), and the hydrophobic amino acids tyrosine (12.4%) and tryptophan (16.40%). EYGF-33 contained hydrophobic amino acids such as alanine (7.8%), leucine (13.8%), and tryptophan (9.4%), and positively charged lysine (8.3%) and arginine (11.8%). The predominant sequences in the fractions related to antioxidant activity were identified by LC-MS as follows: EYGF-23 (TMFPSA, WIHNENQGF and WYGPD); EYGF-33 (MPVHTDAD, KLSDW and YPSPV). The activity exhibited for each peptide was presented in Figure 7.

**Table 2:** Amino acid composition of EYGF-23, EYGF-33 and EYGF-56 fractions. Data correspond to the means ± SD of three independent experiments.

Amino Acids	EYGF-23 (%)	EYGF-33 (%)	EYGF-56 (%)
Aspartic acid	3.02 ± 0.27	6.22 ± 0.02	3.08 ± 0.33
Glutamic acid	3.41 ± 0.26	6.55 ± 0.014	9.30 ± 0.18
Serine	3.7 ± 0.13	5.77 ± 0.001	3.70 ± 0.22
Glycine	2.60 ± 0.11	5.70 ± 0.015	3.18 ± 0.45
Histidine	1.91 ± 0.26	1.21 ± 0.11	0.87 ± 0.57
Arginine	3.82 ±0.12	11.80 ± 0.001	16.25 ±0.53
Threonine	1.101 ± 0.04	0.01 ± 0.2	0.52 ± 0.02
Alanine	2.4 ± 0.14	7.80 ± 0.05	1.75 ± 0.32
Proline	8.01 ± 0.07	5.12 ± 0.006	1.68 ± 0.15
Tyrosine	12.40 ± 0.16	5.01 ± 0.011	1.51 ± 0.26
Valine	5.30 ± 0.23	6.71 ± 0.003	2.76 ± 0.52
Methionine	2.72 ± 0.17	0.03 ± 0.021	3.15± 0.22
Cysteine	0.90± 0.17	0.04 ± 0.13	0.99 ± 0.24
Isoleucine	3.12 ± 0.05	0.10 ± 0.09	1.28 ± 0.19
Leucine	5.7 ± 0.39	13.82 ± 0.013	21.64 ± 0.25
Phenylalanine	2.04 ± 0.10	1.05 ± 0.17	2.98 ± 0.24
Tryptophan	16.40 ± 0.07	9.44 ± 0.016	14.25 ± 0.29
Lysine	11.03 ± 0.02	8.27 ± 0.003	8.05 ± 0.91

The presence of hydrophobic amino acids such tryptophan and tyrosine in EYGF-23 and EYGF-33 may play a role in increasing the interaction between peptides and fatty acids. Moreover, the presence of proline in EYGF-23 with its unique cyclic structure can also enhance the scavenging properties of the peptide. The content of amino acids, their sequence in the isolated peptide and peptide size, all play an important role in determining the activity of a peptide [6]. It is well known that the presence of hydrophobic amino acids in protein hydrolysates or peptides elevates their antioxidant activity by increasing peptide solubility in a lipid system [41]. Chen *et al.* [33,37] illustrated that the presence of hydrophobic moieties valine and leucine at the N-terminus, in an antioxidant peptide was important to obtain access to hydrophobic targets. Luo *et al.* [42] isolated a peptide with the sequence leucine–aspartate–lysine from *Sphyrna lewini* muscle protein, which exhibited powerful scavenging activity.

Saeed *et al.* [43] suggested that, due to the ability of tyrosine to donate hydrogen, it was preferentially oxidized by radicals to protect lipid systems from oxidation. Tryptophan is also an important amino acid and removal of the labile hydrogen attached to the nitrogen of its indole ring produces a free radical that is easily stabilized due to electron delocalization, thereby allowing tryptophan to break the free radical chain reactions and stop the oxidation process. Me *et al.* [44] demonstrated that the presence of a tryptophan at the C-terminus of isolated buckwheat peptides may be responsible for its radical scavenging activity. Therefore, the antioxidant activity of EYGF-23 and EYGF-33 is likely to be related to their amino acid composition.

**Figure 8:**
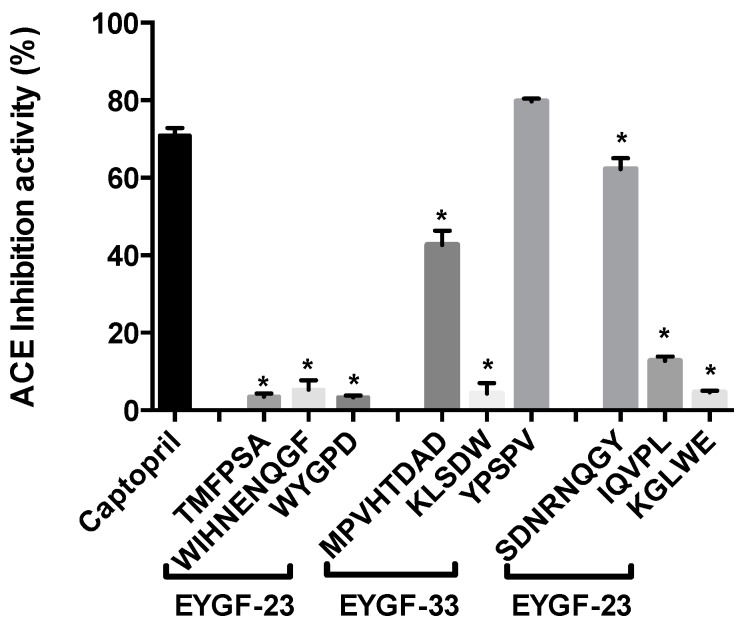
ACE inhibitory activity (%) of selected peptides. The activity of 10 mg/mL for each peptide to inhibit ACE was measured by HPLC. Captopril (0.5 mg/mL) was used as a positive control. Data correspond to the means ± SD of three independent experiments. ANOVA was performed in Graphpad Prism version 6.0 followed by Dunnett’s multiple comparisons test. The result was considered statistically significant *versus* captopril (* = *p* < 0.05).

EYGF-56 with ACE inhibitory activity, contained a high proportion of leucine (21.6%), positively charged arginine (16.3%) and lysine (8.1%), and aromatic tryptophan (14.3%) and negatively charged glutamic acid (9.3%); the main sequences were identified as SDNRNQGY, IQVPL and KGLWE. The SDNRNQGY peptide (10 mg/mL) had ACE inhibitory activity that was not significantly different from that of the positive control captopril (0.5 mg/mL). In addition, YPSPV (EYGF-33) (10 mg/mL) had higher ACE inhibitory activity compared with captopril (0.5 mg/mL) as shown in (Figure 8).

The structure–activity relationship of ACE inhibitory peptides is highly influenced by the sequence of the tripeptide position in the C-terminal that is known to play an important role in the competitive binding of peptide to the active site of ACE [45]. Competitive binding is also affected by the presence of aromatic amino acid at the C-terminal and branched-chain aliphatic amino acids at the N-terminal [28]. In this research, the presence of positively charged arginine and lysine in EYGF-56, as well as the presence of hydrophobic tryptophan and leucine may be responsible for the high activity shown by the fraction. Li *et al.* [45] also found that the presence of hydrophobic amino acids increased ACE inhibitory activity. The hydrophobic and hydrophilic properties of the selected peptides enhanced the stabilization of complex formed by hydrogen bonding and electrostatic interaction between the peptide and the active site of the enzyme [28].

The interaction between ACE inhibitors and the Zn^2+^ in the active site of the enzyme plays a crucial role in enzyme inhibition. Pan *et al.* [28] suggested that leucine and the Zn^2+^ ion are linked via the carbonyl group of leucine and this coordination is responsible for the enzyme inhibition activity. The presence of leucine in EYGF-56 may contribute to the inhibition activity shown in the current study. The ACE inhibitory activity of these peptides may be due to the presence of a positive charge on ε-amino of lysine, or the guanidino group of arginine at the C-terminal of the isolated peptide [46].

## 3. Experimental Section

### 3.1. Protein Extraction and Quantification

The method used was in accordance with Sakanaka *et al.* [21] with some modifications that are detailed below. Briefly, eggs were broken and the yolk was separated from the albumen using filter paper and forceps. In order to remove lipids, egg yolk was defatted using hexane and 69% ethanol alternatively, several times with slow agitation. The ratio of yolk to solvent was 1:3 (*v*/*v*) and 1:5 (*v*/*v*) for 69% ethanol and hexane, respectively. The isolated protein was filtered and then stored at −80 °C for further study. The Kjeldahl method (N × 6.25) AOAC [47] was used to quantify the protein content of egg yolk before and after extraction.

### 3.2. Preparation of Protein Hydrolysates by Enzymatic Digestion

The method of Magias *et al.* [12] was used. Defatted yolk protein powder prepared in the laboratory (Section 3.1) was homogenized with milli-Q (Merck Millipore, Darmstadt, Germany) water (1:2.5 *w*/*v*) for approximately 3–5 min using a magnetic stirrer. The pH of the mixture was adjusted to pH 2.0 with 1 M HCl. Pepsin was added to the substrate in the ratio 1:35 (*w*/*w*) and the suspension was incubated at 37 °C with continuous stirring. After 1 h, the pH was adjusted to pH 7.5 using 1 M NaOH before adding the pancreatin to the mixture in the ratio 1:25 (*w*/*w*). The mixture was incubated again at 37 °C for 2 h with continuous stirring. After the completion of the digestion, the mixture was submerged in boiling water for 20 min to inactivate the enzyme and terminate the digestion process. The hydrolyzed protein was centrifuged at 12,000× *g* for 15 min.

### 3.3. Fractionation of Egg Yolk Hydrolysate by Ultrafiltration

The resultant hydrolysate was fractionated using ultrafiltration membrane cartridges with molecular weight cut-offs of 10 followed by 5 and then 2 kDa. After passage through each membrane, resultant aliquots were centrifuged at 3500 × *g* for 30 min; the resultant fractions were lyophilized and stored at −80 °C for further studies.

### 3.4. Purification Using Gel Filtration Chromatography

Samples that exhibited the highest activity were fractionated by gel filtration chromatography using an Ultimate 3000 HPLC (Thermo Fisher Scientific Inc., Loughborough, UK). A Sephadex G-25 gel filtration column (Amersham Pharmacia Biotech, Little Chalfont, Buckinghamshire, UK) (2.5 cm × 90 cm) was equilibrated with a 50 mM sodium phosphate buffer (pH 7.0). The sample (240 mg), dissolved in 6 mL of a 50 mM sodium phosphate buffer (pH 7.0), was injected onto the column and eluted with the same buffer for 10 h at a constant flow rate of 1 mL/min. Resultant fractions were collected, pooled from 15 different chromatogaphy runs, and lyophilized to be ready for further study.

### 3.5. Evaluation of Lipid Oxidation in Linoleic Acid Model System

The inhibition of lipid peroxidation by the EYPH fractions was measured using linoleic acid oxidized as described below [48]. The degree of oxidation was measured using (FTC) and (TBARS) methods. In order to prepare the reaction mixture, 50 mg of each sample was dissolved individually with 10 mL absolute ethanol, 0.13 mL linoleic acid, 4.87 mL distilled water and 10 mL 50 mM sodium phosphate buffer (pH 7). Samples were homogenized by a sonicator (Labsonic^®^ M, B. Braun Biotech International, Melsungen, Germany). The tubes were then sealed tightly with silicone rubber caps and kept in the dark at 40 °C in an oven. Aliquots for FTC and TBARS were taken from these samples daily to measure the activity over 7 days. A negative control was prepared in the same manner using distilled water in place of egg yolk hydrolysates. The commercial antioxidants, 0.2 mg/mL of BHT and trolox were also used as positive controls.

#### 3.5.1. Ferric Thiocyanate (FTC) for Peroxide Formation

At 24 h intervals, 100 μL aliquots were drawn from each of the reaction mixtures with a micro syringe and added to a test tube containing 4.7 mL 75% ethanol (*v*/*v*) and 100 μL 30% ammonium thiocyanate (*w*/*v*). Then, 100 μL 20 mM ferrous chloride solution was added. After exactly 3 min, the absorbance of the resulting ferric thiocyanate solution was read at 500 nm using a spectrophotometer (Kontron Instrument UNIKON 860, Cambridge, UK).

#### 3.5.2. Determination of Thiobarbituric Acid Reactive Species (TBARS)

At 24 h intervals, a 50 μL aliquot was drawn from each of the previous reaction mixtures with a microsyringe and added to test tubes containing 0.8 mL of distilled water, 0.2 mL of 8.1% sodium dodecyl sulphate (*w*/*v*), (1.5 mL) 20% (*w*/*v*) acetic acid (pH 3.5) and 1.5 mL 0.8% 2-thiobarbituric acid solution in water (*w*/*v*). The mixture was heated at 100 °C for 60 min. After cooling, the mixture was centrifuged at 4300× *g* for 10 min. The absorbance of the upper layer was measured at 532 nm using a spectrophotometer (Kontron Instrument UNIKON 860) Malonaldehyde standard curve was prepared using 1,1,3,3-tetramethoxypropane and TBARS were expressed as μg of malonaldehyde/mL. The percentage (%) of lipid oxidation inhibition was calculated according to the following formula:

$$\text{Lipid oxidation inhibition }(\%)=\frac{\text{Absorbance of control }-\text{ Absorbance of sample}}{\text{Absorbance of control}}\times 100$$

### 3.6. Measurement of Antioxidant Activity

#### 3.6.1. Scavenging Activity on DPPH (1,1-Diphenyl-2-Picrylhydrazyl) Radical

The method used was in accordance with Bersuder *et al.* [49] using the synthetic free radical DPPH. Briefly, a volume of 500 μL of selected fractions at different concentrations (0.5, 1, 5, 10 and 20 mg/mL) was mixed with 500 μL of 99.5% ethanol and 125 μL of 0.02% *w*/*v* DPPH prepared previously in 99.5% ethanol. The mixture was shaken vigorously then kept at room temperature in the dark. After 60 min, the color reduction of the DPPH substrate was measured at an absorbance of 517 nm. Trolox and BHT (both used at concentration 0.2 mg/mL) were also tested as positive controls for comparison. The DPPH radical scavenging activity was calculated as follows:

$$\text{Radical scavenging activity }(\%)=\frac{A_{C}-A_{S}}{A_{C}}\times 100$$

where *A_C_* is the absorbance for negative control using 500 μL deionized water instead of sample, *A_S_* is the absorbance of the sample or positive controls (trolox/BHT). A lower absorbance of the reaction mixture indicated a higher DPPH radical-scavenging activity.

#### 3.6.2. Hydroxyl Radical Scavenging Activity (HRSA) Assay

The HRSA assay was performed as described by Wu *et al.* [50]. The reaction mixture consisted of 0.1 mL of 10 mM FeSO_4_, 0.1 mL of 10 mM EDTA, 0.5 mL of 10 mM α-deoxyribose, 0.9 mL sodium phosphate buffer pH 7.4, and 0.2 mL of peptides at different concentrations (0.5, 1, 5, 10 and 20 mg/mL). All reagents were then thoroughly mixed in a tube and 0.2 mL of 10 mM hydrogen peroxide was added. The reaction mixture was then incubated at 37 °C for 1 h. Following incubation, 1 mL of 2.8% TCA and 1 mL of 1.0% TBA were added to the test tubes, and the contents boiled for 15 min. After cooling at room temperature, the absorbance of the mixture was measured at 532 nm. Trolox and BHT (both used at concentration 0.2 mg/mL) were also tested as positive controls for comparison. The HRSA was evaluated as a percentage of α-deoxyribose oxidation inhibition by the hydroxyl radical and calculated as follows:

$$\text{HRSA }(\%)=\frac{A_{C}-A_{S}}{A_{C}}\times 100$$

where *A_C_* is the absorbance for negative control using 500 μL deionized water instead of sample, *A_S_* is the absorbance of the sample or positive controls (trolox/BHT).

#### 3.6.3. Superoxide Anion Scavenging Activity (Pyrogallol)

In this assay, the alkaline condition enhances the autoxidation of pyrogallol to produce a superoxide anion, which, in turn, accelerates the autoxidation process by autocatalysis. The autoxidation of a pyrogallol method described by Marklund and Marklund [51] was applied to measure the superoxide anion scavenging power of egg yolk peptides. The reaction mixture consisted of 1.0 mL of peptides sample at different concentrations (0.5, 0.1, 5.0, 10 and 20 mg/mL) and 1.8 mL of 50 mM Tris-HCl buffer, previously adjusted to pH 8.2. The mixture was incubated at 25 °C for 20 min. Then, 40 μL of 45 mM pyrogallol (prepared previously in 10 mM HCl) was added. The absorbance of the reaction mixture was measured at 320 nm immediately after adding pyrogallol at 1 min intervals up to 4 min. Trolox and BHT (both used at a concentration of 0.2 mg/mL) were also tested as positive controls for comparison. The O_2_**^•−^** scavenging activity was calculated as follows:

$$\text{The }{{\text{O}}_{2}}^{•-}\text{ scavenging activity }\left(\%\right)=\frac{\Delta A_{C}-\Delta A_{S}}{\Delta A_{C}}\times 100$$

where (Δ*A_C_*) is the autoxidation rate of pyrogallol in negative control samples, measured using 1.0 mL deionized water, (Δ*A_S_*) is the oxidation rate of pyrogallol for samples or positive controls (trolox/BHT).

#### 3.6.4. Ferrous Chelating Activity Assay

The activity of the peptide to chelate ferrous Fe^2+^ ions was measured according to the method of Ebrahimzadeh *et al.* [52]. The reaction mixture contained 0.5 mL of isolated peptides at different concentrations (0.5, 1, 5, 10 and 20 mg/mL), 1.6 mL deionized water and 0.05 mL of 2 mM FeCl_2_. After 30 s, 0.1 mL of 5 mM ferrozine was added. After 10 min at room temperature, the absorbance of the Fe^2+^-ferrozine complex was measured at 563 nm. EDTA (0.2 mg/mL) was also tested as a positive control for comparison. The activity of the peptide to chelate Fe^2+^ was calculated as:

$$\text{Chelating ability }\left(\%\right)\;=\frac{A_{C}-A_{S}}{A_{C}}\times 100$$

where *A_C_* is the absorbance of the control, using 0.5 mL deionized water instead of sample, *A_S_* is the absorbance of the sample or positive control EDTA.

### 3.7. Determination of Angiotensin Converting Enzyme Inhibitory Activity

The measurement of ACE inhibitory activity was conducted using HPLC in accordance with a modified method of Wu *et al.* [53]. All samples were prepared with 100 mM borate buffer, containing 300 mM NaCl at pH = 8.3. The total reaction volume was 70 μL, consisting of 50 μL of 2.17 mM hippuryl-histidyl-leucine (HHL), 10 μL of peptide fractions and 10 μL of 2 mU angiotensin converting enzyme (ACE). The peptide fractions and HHL were mixed and maintained at 37 °C for 10 min in 2 mL polyethylene microcentrifuge tubes. ACE was maintained at 37 °C for 10 min before the two solutions were combined. The combined mixture was incubated at 37 °C for 30 min with continuous agitation. In order to terminate the reaction, 85 μL of 1 M HCl was added. Samples were analyzed on a C18 column (3.0 mm × 150 mm, 5 μm, phenomenex) and hippuric acid (HA) and HHL were detected at 228 nm using HPLC system (Ultimate 3000 HPLC, Thermo Fisher Scientific Inc., Loughborough, UK). Data were analyzed using integration software (Chromeleon, Thermo Fisher Scientific Inc., Loughborough, UK). The column was eluted at a rate of 1 mL/min with a two solvent system: (A) 0.05% trifluoroacetic acid (TFA) in water and (B) 0.05% TFA in acetonitrile, with a 5%–60% acetonitrile gradient for the first 10 min, maintained for 2 min at 60% acetonitrile, then returned to 5% acetonitrile for 1 min. This was followed by isocratic elution for 4 min at the constant flow rate of 1 mL/min. The ACE inhibitory activity was calculated as follows:

$$\text{ACE inhibitory activity }\left(\%\right)\;=\frac{\text{C}-\text{S}}{\text{C}-\text{B}}\times 100$$

where C is the peak area of control (buffer added instead of test sample), B the peak area of the reaction blank (without ACE and sample), and S is the peak area in the presence of a sample or captopril.

### 3.8. Amino Acid Composition of Isolated Egg Yolk Peptide

Amino acid content was determined for samples exhibiting high antioxidant activity after the gel filtration process, according to Badii and Howell [54]. Each sample (5 mg) was dissolved in 5 mL 6 N HCl in dark screw-cap tubes. Oxygen was expelled from the samples using a nitrogen pump before incubation in oven at 110 °C for 24 h. The hydrolyzed samples were then subjected to a derivatization step followed by analysis using HPLC (Ultimate 3000 HPLC, Thermo Fisher Scientific Inc., Loughborough, UK). Amino acids were separated on a C18 reverse-phase column (3.9 mm × 150 mm, 5 um particle size) using gradient mobile phase consisting of two eluents; A and B. Eluent A was composed of (0.22 M sodium acetate buffer containing 0.05% (*v*/*v*) TEA, adjusted to pH 6.2 using glacial acetic acid) and eluent B was composed of acetonitrile: water (60:40 *v*/*v*).

### 3.9. Peptide Sequencing by LC/ESI MS/MS

Peptides (500 ng to 2 μg) were analyzed by LC/ESI MS/MS with a Thermo Scientific Easy-nLC II (Thermo Scientific, Waltham, MA, USA) nano HPLC system coupled to a hybrid Orbitrap Elite ETD (Thermo Scientific, Waltham, MA, USA) mass spectrometer using an instrument configuration as described [55]. In-line de-salting (except for direct inject runs) was accomplished using a reversed-phase trap column (100 μm × 20 mm) packed with Magic C_18_AQ (5-μm 200 Å resin; Michrom Bioresources, Auburn, CA, USA) followed by peptide separations on a reversed-phase column (75 μm × 250 mm) packed with Magic C_18_AQ (5-μm 100 Å resin; Michrom Bioresources, Auburn, CA, USA) directly mounted on the electrospray ion source. A 60-min gradient from 7% to 35% acetonitrile in 0.1% formic acid at a flow rate of 400 nL/min was used for chromatographic separations. The heated capillary temperature was set to 300 °C and a spray voltage of 2000 V was applied to the electrospray tip. The Orbitrap Elite instrument was operated in the data-dependent mode, switching automatically between MS survey scans in the Orbitrap (AGC target value 1,000,000, resolution 240,000, and injection time 250 milliseconds) with MS/MS spectra acquisition in the linear ion-trap (AGC target value of 10,000 and injection time 100 milliseconds). The 20 most intense ions from the Fourier-transform (FT) full scan were selected for fragmentation in the linear ion trap by collision-induced dissociation with a normalized collision energy of 30%. Selected ions were dynamically excluded for 45 s with a list size of 500 and exclusion mass-by-mass width ±10 ppm. The *m*/*z* range for MS1 was 400 to 1800 (except for the direct injection which was set to *m*/*z* 300 to 1600). Charge state selection was enabled where +2 and +3 were selected for MS/MS or +1, +2 and +3 were selected for MS/MS.

Data analysis was performed using Proteome Discoverer 1.4 (Thermo Scientific, San Jose, CA, USA). The data were searched against a Uniprot Swiss Prot database that included common contaminants. Searches were performed with no enzyme, minimum mass 150 Da and maximum mass 5000 Da. The minimum peptide length was set to 4 with maximum peptide length 40. The precursor ion tolerance was set to 10 ppm and the fragment ion tolerance was set to 0.6 Da. Variable modifications included oxidation on methionine and proline (+15.995 Da). Sequest HT was used for database searching and fixed Value PSM Validator was used for scoring (Thermo Scientific, San Jose, CA, USA).

### 3.10. Peptide synthesis

Based on the above analysis, selected peptides were synthesized by Cambridge Research Biochemicals, UK for further tests.

### 3.11. Data Analysis

Each experimental condition was tested in triplicate and the experiment repeated three times on separate occasions to ensure reproducibility. Results were analyzed using GraphPad Prism software version 6.0 (GraphPad Software Inc., La Jolla, CA, USA). Statistical analysis comparisons were made by one-way analysis of variance (ANOVA) followed by Dunnett’s multiple comparisons test. Details of the statistical test used are provided in the figure legends of presented results. *p* < 0.05 was considered statistically significant. Data were presented as means ± standard deviation (SD).

## 4. Conclusions

Novel bioactive EYGF-23, EYGF-33 and EYGF-56 fractions were successfully isolated and purified from egg yolk protein, a by-product of lecithin production. EYGF-23 and EYGF-33 inhibited oxidation induced in a linoleic acid oxidizing model system by a variety of mechanisms (radical scavenging and metal chelating) indicating their potential to function in diverse oxidizing environments. The antioxidant activity was related to the high content of hydrophobic amino acids and the peptides with sequences TMFPSA, WIHNENQGF, WYGPD (EYGF-23) and MPVHTDAD, KLSDW, YPSPV (EYGF-33). Additionally, fraction EYGF-56 exhibited high ACE inhibition activity (69.2%) and IC_50_ of 3.35 mg/mL. The presence of predominantly leucine, which can bind to Zn^2+^ at the enzyme active site, as well as positively charged lysine and arginine and hydrophobic tryptophan in EYGF-56 (peptide sequences SDNRNQGY, IQVPL and KGLWE) may be responsible for the ACE inhibitory activity with SDNRNQGY (10 mg/mL) showing ACE inhibitory activity that was not significantly different from captopril (0.5 mg/mL). Although these findings indicate a high potential for producing bioactive peptides from egg yolk, clinical studies are needed before they can be used for pharmaceutical applications and functional foods.
